# The outcome in pediatric acute myeloblastic leukemia; results of the first-line treatment and contribution of hematopoietic stem cell transplantation to survival of relapsed patients

**DOI:** 10.55730/1300-0144.5806

**Published:** 2023-12-11

**Authors:** Nazan SARPER, Intzi TACHSIN, Sema AYLAN GELEN, Emine ZENGİN

**Affiliations:** Department of Pediatrics, Medical Faculty, Kocaeli University, Kocaeli, Turkiye

**Keywords:** Acute myeloblastic leukemia, pediatric, childhood, acute leukemia

## Abstract

**Background/aim:**

To analyze the long-term outcome of pediatric patients with acute myeloblastic leukemia.

**Materials and methods:**

Data from 69 patients 0–18 years of age diagnosed between December 2001 and October 2019 were analyzed in April 2023. Patients received MRC-AML10 chemotherapy (2ADE+MACE+MidAC). No maintenance chemotherapy or preventive cranial radiotherapy was administered. Twelve patients with Down syndrome and 15 patients with promyelocytic leukemia were in the cohort. Patients with Down syndrome received reduced chemotherapy (cumulative anthracycline 420 mg/m^2^, cytarabine 3.4 g/m^2^, etoposide 1400 mg/m^2^). ATRA was added to chemotherapy in promyelocytic leukemia.

**Results:**

Four patients (5.8%) died in the induction (two typhlitis, one intracranial hemorrhage, and one resistant disease). The complete remission rate of 66 patients was 87.8%. There was one death due to cardiotoxicity. Total infection-related deaths were 7.2%. Seven patients with high-risk criteria and one with resistant disease underwent hematopoietic stem cell transplantation (HSCT) following the first-line treatment. All seven patients in remission were alive and disease-free. The relapse rate was 34.4% (n = 21). Four patients developing marrow relapse were disease-free in the second remission after salvage and maintenance chemotherapy. Thirteen patients (18.84%) underwent HSCT in the second remission and 8 are alive and disease-free. The mean follow-up period of patients from diagnosis was 185 ± 13 months. Thirty-four patients (49.2%) were alive and disease-free in the first remission whereas another two patients in the first remission developed secondary malignancy. In good, standard, and poor risk groups, event-free survival (EFS) rates were 68.2%, 52.9%, and 10%, and overall survival (OS) rates were 86.4%, 79.4%, and 20%, respectively. Fifteen years of EFS and OS of the whole cohort were 49.3% and 69.6%, respectively.

**Conclusion:**

When compared with national data and multicenter studies of developed countries, survival rates were acceptable.

## 1. Introduction

Acute myeloblastic leukemia (AML) accounts for only 20% of acute leukemias in childhood. The survival rates continue to improve in studies of large groups such as the International Berlin Frankfurt Munster study group (I-BFM-SG), Children’s Oncology Group (COG), and the Medical Research Council (MRC) [1–3]. This is achieved by the intensification of chemotherapy-mainly increasing cytarabine doses, better supportive care, and implementation of the allogeneic hematopoietic stem cell transplantation (HSCT) of high-risk, resistant, or relapsed patients. Better supportive care decreases mortality due to bleeding, leukostasis, or infections [1].

About 35% of pediatric AML patients relapse [3]. The overall survival (OS) after relapse at 5 years in AML-BFM protocols (2004–2017) and COG protocols (2006–2018) was 42% ± 4% and 35% ± 2%, respectively. Donor availability and experience in HSCT increased and transplant-related mortality decreased in relapsed patients in the last decades [1]. One-year maintenance treatment in AML was used by the BFM group but their survival rates were not higher than other groups and it was found of no benefit in a study [4]. Preventive cranial irradiation (RT) was also used by the BFM group but it was stopped in May 2009 after a randomized study comparing 12 Gy with 18 Gy [5].

In Türkiye, prospective multicenter studies were not conducted in childhood AML. Published reports of single centers were also very limited [6]. In this retrospective analysis, we report treatment results in 69 pediatric patients with AML treated on MRC AML 10, with four blocks of intensive chemotherapy. No preventive cranial RT or maintenance was employed in the first-line treatment. National pediatric data from the thesis were also reviewed and compared with the outcomes of the present study [7–11].

## 2. Material and methods

Data of all previously untreated 0–18 years-old patients with AML, diagnosed between December 2001 and October 2019 were analyzed in April 2023 retrospectively from paper files and digital records of the center. There were also patients with Down syndrome, promyelocytic leukemia, and myelodysplastic syndrome (MDS)/AML in the cohort. Routine morphological, immunohistochemical, flow-cytometric, and genetic evaluations of bone marrows were performed at the presentation. Patients received MRC-AML10 without any randomization. Two induction chemotherapy courses (ADE-I 5 + 10 + 3 and ADE-II 5 + 8 + 3) and two consolidation courses (MACE 5 + 5 + 5 and MidAC 5 + 3) were administered with cumulative anthracycline doses of 550 mg/m^2^, cytarabine 10.6 g/m^2^, and etoposide 1500 mg/m^2^. However due to the unavailability of amsacrine MACE course was modified to the ICE course (Idarubicin 20 mg/m^2^, cytarabine 1 g/m^2^, etoposide 500 mg/m^2^) in most of the patients. In infants, chemotherapy doses were reduced by 25%. Age-adjusted triple intrathecal chemotherapies (methotrexate + cytarabine + dexamethasone/prednisolone) were administered in each course for central nervous system (CNS) prophylaxis [3]. Preventive cranial RT and maintenance chemotherapy were not introduced in the first-line treatment. No patient underwent leukapheresis or dialysis for prophylaxis or treatment of tumor lysis syndrome [12].

In patients with promyelocytic leukemia all-trans retinoic acid (ATRA) 25–40 mg/m^2^/day was also added to chemotherapy. It was administered until remission and in the first 14 days of the following chemotherapy courses.

In patients with Down syndrome reduced chemotherapy doses were administered. Two induction chemotherapy courses (ADE-I 7 + 3 + 5, ADE-II 6 + 3 + 5) and two consolidation courses (ICE 2 + 5 + 5 and MidAC 5 + 3) were used with cumulative anthracycline doses of 420 mg/m^2^, cytarabine 3.4 g/m^2^ and etoposide 1400 mg/m^2^ (1 mg/m^2^ mitoxantrone = 1 mg/m^2^ idarubicin = 5 mg/m^2^ daunorubicin were toxicity equivalence ratios).

High-risk patients or patients in partial remission received HSCT in the first remission if an appropriate donor was available. Fludarabine, cytarabine, G-CSF, idarubicin (FLAG-IDA), and FLAG chemotherapy courses were administered to patients with the resistant disease after induction II or as salvage chemotherapy after relapse. Cotrimaxazol was used for P*neumocystis jirovecii* prophylaxis thrice weekly. From July 2016, voriconazole and ciprofloxacin prophylaxis were administered in neutropenic periods.

Definitions and statistics: After the assessment of bone marrow at the end of induction I, patients fall into three remission classes.

Complete remission (CR) was defined as a bone marrow containing <5% leukemic cells with regeneration of hematopoietic cells (M1 bone marrow).

Partial remission (PR) was defined as a bone marrow between 5% and 20% leukemic cells with regeneration of hematopoietic cells (M2 bone marrow).

Resistant disease (RD) was defined as bone marrow containing >20% leukemic cells (M3 bone marrow).

In MRC-AML10, risk groups were defined as follows:

Good risk: Any patient with favorable karyotypic abnormalities-i.e. t(8;21), t(15;17), and inv(16) irrespective of marrow status after course I or presence of other karyotypic abnormalities.

Standard risk: Any patient not in either good or poor risk groups.

Poor risk: Any patient with more than 20% blasts in the bone marrow performed after course I and without favorable karyotypic abnormalities.

Induction death: Death within 42 days of diagnosis due to any cause.

Event-free survival (EFS) was calculated from the date of diagnosis to the date of the first event (relapse or death from any cause).

OS was calculated from the date of diagnosis to death from any cause.

Relapse rate (RR): For patients achieving remission probability of relapse.

Descriptive statistics were performed for the median, mean, and percentage calculations. For the comparison of groups Mann-Whitney U test and Kruskal Wallis test (p-value < 0.05 was significant) and for survival analysis Kaplan-Meier method was used. SPSS for Windows 13.0 was used for the analysis.

## 3. Results

**Figure 1:**
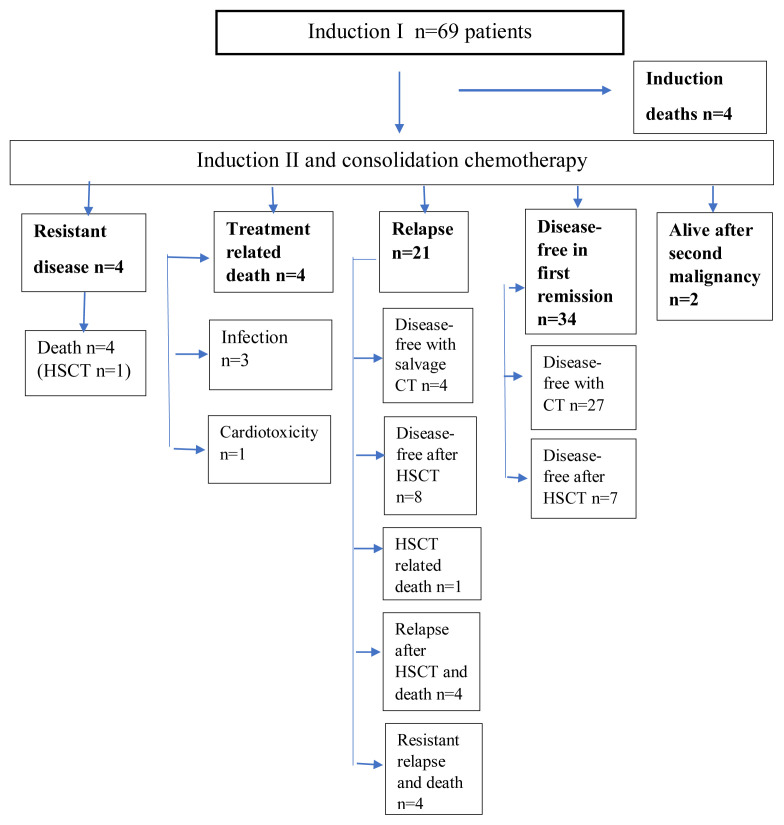
Flow-chart of pediatric patients with acute myeloblastic leukemia.

There was no treatment abandonment. MRC-AML10 protocol was administered to 69 patients with a median age of 9.0 years (0.6–17.5). Characteristics and outcomes of pediatric patients with AML are shown in Table 1. There was male predominance in this cohort (M/F = 44/25 = 1.8/1). The median white blood cell count was 11.007/μL (200–400,000) at presentation. Distribution of patients’ FAB subtypes: M1, M2, M3, M4, M5, M6 and M7 were 5.8% (n = 4), 21.6% (n = 15), 21.6% (n = 15), 10.1% (n = 7), 11.6% (n = 8), 5.8% (n = 4) and 17.4% (n = 12) respectively. FAB type was undefined in 4(5.8%) patients. Five patients (7.2%) transformed to AML from MDS. No patient showed CNS involvement at the presentation. The causes of four induction deaths were typhlitis in two patients, resistant disease in one patient, and intracerebral hemorrhage in one patient with promyelocytic leukemia and hyperleukocytosis. Flow-chart of patients is shown in Figure 1. Three of these patients died before the postinduction remission assessment of bone marrow. In the remaining 66 patients, there was M1, M2, and M3 marrow in 52, 2, and 12 patients respectively after induction I. Seven patients achieved remission after subsequent courses. As a result, remission was achieved in 52 patients (78.7%) after induction I and in 59 patients (89.3%) after induction II. One patient in the first remission died due to cardiotoxicity during consolidation. She developed pericardial tamponade and died in the third month of treatment. There was no infection-related death in 16 patients on voriconazole plus ciprofloxacin prophylaxis whereas there were 9.4% (5/53) infection-related deaths in other patients. Total infection-related deaths were 7.2% (5/69). Seven of the patients with high-risk criteria underwent HSCT in the first remission and they were all disease-free. One resistant patient underwent HSCT without achieving remission; she survived for three years but then relapsed and died. Thirty-four patients (49.3%) were alive and disease-free in the first remission.

Twenty-one patients developed relapses. The first relapse was in the median 14th month (7–76 months). There were 19 isolated bone marrow relapses one isolated testis and one combined bone marrow plus CNS relapse. Four patients who developed marrow relapse after MRC-AML 10 chemotherapy were disease-free in the second remission after salvage chemotherapy and maintenance chemotherapy without HSCT. One of these patients had promyelocytic leukemia. Two of them were standard risk and one was a high-risk patient. Relapses developed, at 15, 15, 30, and 78 months of diagnosis and all are long-term survivors. High-risk patients developed late relapse. Out of 21 relapsed patients, 13 (61.9%) underwent HSCT in the second remission and eight were alive and disease-free. In the first HSCTs, matched-sibling donors, matched-unrelated donors, and haploidentical donors were used in 10 (47.6 %), 7 (33.3 %), and 4 (19 %) transplants, respectively.

Out of 12 patients with Down syndrome, three transformed from MDS. Two of the patients suffered from induction deaths. Ten patients (83.3%) with Down syndrome were disease-free in the first remission.

There were 25 patients with favorable genetic features and 22(88%) of them were alive and disease-free. Out of 15 patients with promyelocytic morphology, only one patient died due to cerebral hemorrhage in the induction; 13(86.6%) were disease-free in the first remission and one patient in the second remission. Out of nine patients with t(8;21) one died due to infection. Three patients relapsed and underwent HSCT. One patient died of HSCT-related complications. As a result, seven patients (77.7%) with t(8;21) were alive, five patients were in the first and two patients in the second remission. One patient with inv(16) is alive in the first remission.

Two patients who were in the first remission, developed secondary malignancy-acute lymphoblastic leukemia in 11.5 years and breast cancer in 16.2 years of diagnosis. Their treatment was given in adult hematology-oncology clinics.

In good, standard, and poor risk groups, EFS rates were 68.2%, 52.9%, and 10%, and OS rates were 86.4%, 79.4%, and 20%, respectively. Fifteen years of EFS and OS of the whole cohort were 49.3% and 69.6%, respectively. EFS and OS of the cohort are shown in Figures 2a, 2b and Figures 3a, 3b.

## 4. Discussion

In our center, MRC-AML 10 chemotherapy was introduced without any randomization, with two induction and two consolidation chemotherapy blocks. In MRC AML-10 no maintenance chemotherapy was administered after its limited value was shown in MRC AML 9 [3]. In the original MRC AML-10 trial, in patients diagnosed between 1988 and 1995, 5-year OS, EFS, and RR were 58%, 49%, and 42%. In MRC AML-12, in patients diagnosed between 1995 and 2002, OS, EFS, and RR were 66%, 56%, and 35%. In Türkiye, pediatric centers accept patients younger than 18 years. In MRC trials, patients younger than 16 years were enrolled but during outcome calculations, patients between 0 and 14 years were included. Patients with MDS and Down syndrome were also excluded from outcome calculations [3]. These differences may influence outcomes. In our study which included patients diagnosed between 2001 and 2019, the mean follow-up period of patients from diagnosis was 185 ± 13 months. Fifteen years, OS, EFS, and RR were 69.6%, 49.3%, and 34.4% which were acceptable. Ten-year EFS was 52.2% because two secondary malignancies developed after ten years.

Outcomes improved in consecutive AML-BFM trials. Between 1987–1992, 5-year OS and EFS were 49% ± 3% and 41% ± 3% and after the introduction of high dose cytarabine/mitoxantrone between 1993–1998, EFS only increased to 50% ± 2%. Between 2011 and 2020, OS increased to 76% ± 4%. Intensification of treatment increased remission rates. Better supportive care decreased deaths due to bleeding and leukostasis from 8.1% in early trials to 2.2%, also infection-related deaths decreased. Relapses did not decrease significantly but salvage chemotherapy and allogeneic HSCT improved OS in relapsed patients. In BFM trials, between 1987 and 1992, 5-year OS after relapse was only 19% ± 4%. OS increased to 45% ± 4% between 2005 and 2010. In these trials, patients 0–18 years of age were enrolled, but patients with secondary leukemia, Down syndrome, or promyelocytic leukemia were excluded from the analysis due to their unique features and treatment [1]. In a retrospective analysis of two large international study groups, (BFM and COG) OS of the patients who underwent postrelapse HSCT was 54% ± 4% [13]. In our cohort also out of 21 relapsed patients, 12 were disease-free (57.1%) after salvage chemotherapy ± HSCT. OS for patients that underwent postrelapse HSCT was 61.5% (8/13). In our cohort, 88.8% (24/27) of patients with Down syndrome plus promyelocytic leukemia were alive and disease-free. When the remaining patients were analyzed 57.1% (24/42) of the patients were alive and disease-free in the 10-year follow-up. The induction death rate was 5.8% (4/69). Postinduction infection-related deaths were 4.3% (3/69) without significant difference in patients on voriconazole plus ciprofloxacin prophylaxis. But only 16 patients received prophylaxis and there was no infection-related death. Two patients with Down syndrome and one patient with promyelocytic leukemia died in the induction. When patients with Down syndrome and promyelocytic leukemia were excluded, the induction death rate would be 2.3% (1/42).

Reports from Turkish centers are shown in Table 2. In these reports, the patients were diagnosed between 1991 and 2018. EFS ranged between 45% and 58.2% and OS ranged between 35.2% and 60%. Induction deaths ranged between 3.5% and 11% and RR ranged between 25.7% and 40.4%. Among these five centers, only one center used the MRC-AML 12 protocol, while others mainly used AML-BFM protocols. Single-center outcomes have lower statistical power due to patient numbers ranging between 36 and 74 compared to multinational studies. Reports reveal that two centers, one from central Anatolia and the other from East Anatolia could not refer any patient to HSCT centers in those years [7,9]. After 2012, access to HSCT and matched-unrelated donors increased. The number of pediatric HSCT centers and their experiences in alternative donor transplants also increased. In our cohort, male patients were predominant (M/F = 1.8/1) similar to the four Turkish centers’ cohorts shown in Table 2. In MRC AML 10 and 12 cohorts also, there was male predominance; 57% and 53% of 0–14 years of age patients were male, respectively [3].

Patients with promyelocytic leukemia were the largest subgroup which was 21.6% of the patients in this cohort. Aksu T et al. reported 17 patients with promyelocytic leukemia, which were 20.5% of their pediatric patients with AML. They were also treated with chemotherapy plus ATRA and two patients died early due to intracranial hemorrhage and septicemia. The early death rate was 12%, and the 5-year OS was 82.5% [6]. In promyelocytic leukemia, intracranial hemorrhage was an important cause of early mortality in contrast to a high remission rate and survival in the first remission. In the MRC AML cohort, only 9% of the patients were in the promyelocytic leukemia subgroup [3].

AML is a heterogeneous disease. Genetic studies predict outcomes and help to plan the treatment strategy for each patient. MRD assessment after induction by multidimensional flow-cytometry allocates patients to HR treatment, or MRD monitoring by molecular markers (fusion genes, mutations) helps to plan HSCT before morphological relapse [14]. Targeted therapies such as FLT3 inhibitors were also added to standard chemotherapy in randomized COG-AML1031 trials. The addition of bortezomib to standard chemotherapy did not improve survival but increased toxicity [15]. Less toxic anthracycline liposomal daunorubicin was tried in AML-BFM 2004 and during induction of relapsed patients with FLAG chemotherapy and showed a better response and less toxicity in some subgroups [16, 17]. International collaborative studies were planned because pediatric AML is rare with an incidence of only seven patients per one million children annually [14].

## Limitations of the study

Comprehensive molecular profiling of leukemia was not available which is predictive for treatment outcome. MRD evaluation by flow cytometry was not also available. Data about second transplants were not analyzed.

## Conclusion

It is suggested that induction death rates, remission rates, RR, survival rates after relapse, and OS rates are acceptable compared to national and international data. Better access to genetic and MRD studies, availability of intensive care units and increasing experience in HSCT will further increase survival rates.

## Figures and Tables

**Figure 2a:**
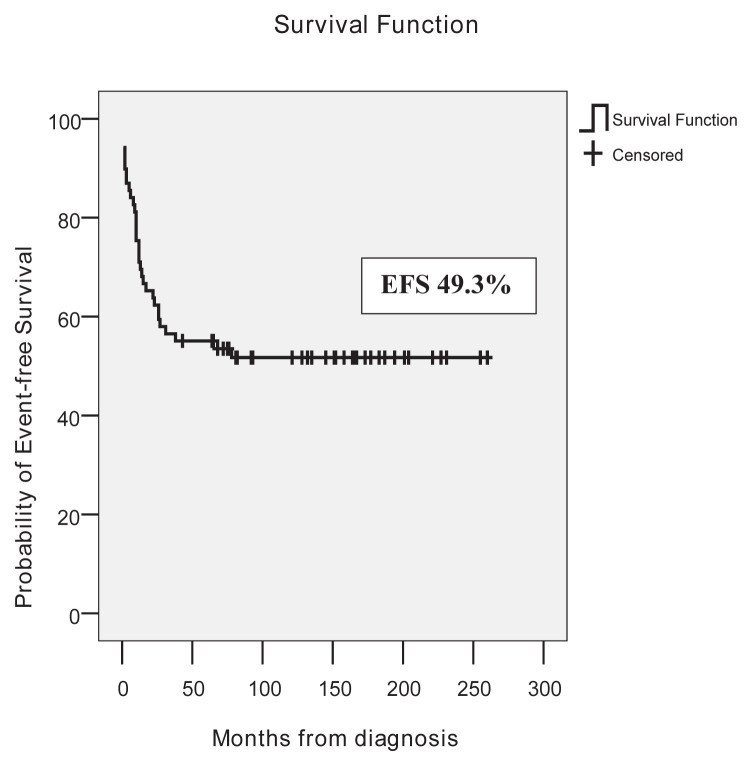
Event-free survival of patients.

**Figure 2b:**
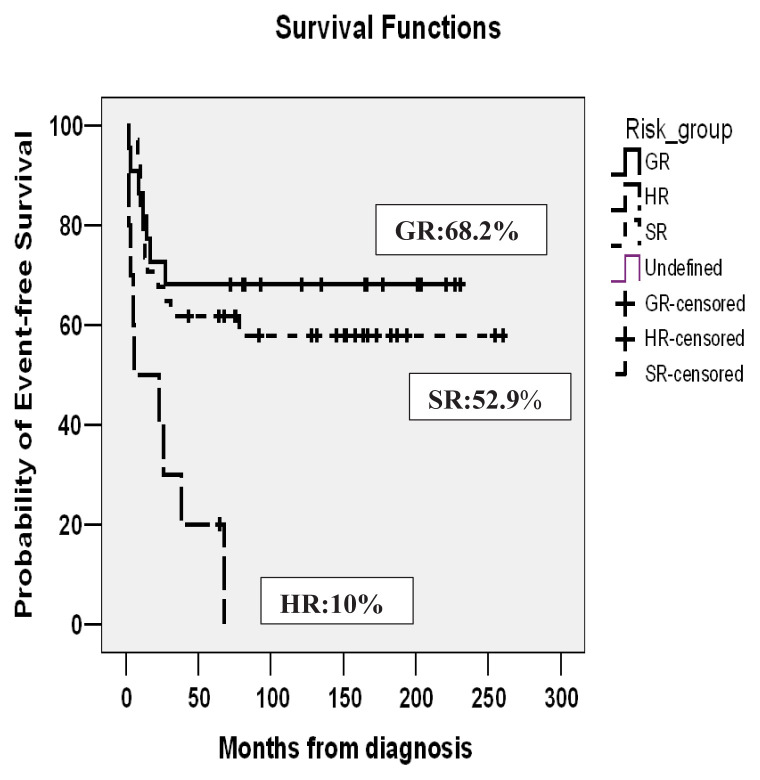
Event-free survival of risk groups.

**Figure 3a:**
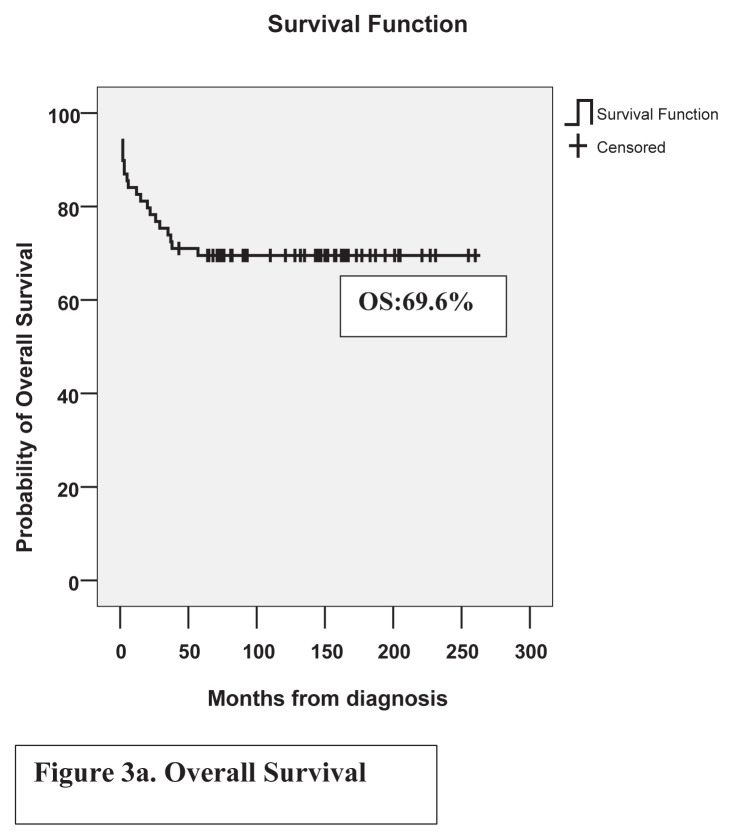
Overall-survival of patients.

**Figure 3b:**
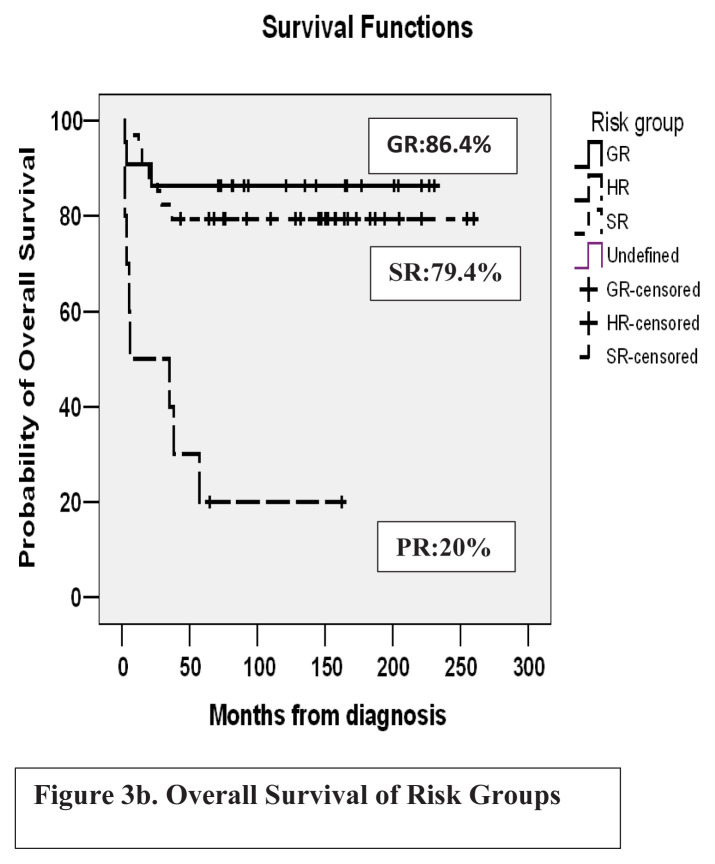
Overall-survival of risk groups.

**Table 1:** Characteristics and outcome of pediatric patients with acute myeloblastic leukemia.

Patient features	n %

Patients with favorable genetics	25 (36.2)
t(15;17)	15 (21.6)
t(8;21)	9 (13.0)
inv(16)	1 (1.4)

Good risk	24 (36.3)
Standard risk	30 (45.4)
Poor risk	12 (18.1)
Death before evaluation	3

Relapse rate	21 (34.4)

Induction deaths	4 (5.8)
Post-induction infections	3 (4.3)
Resistant disease	4 (5.8)
Toxicity related deaths	1 (1.4)
Resistant relapse	4 (5.8)
HSCT related death	1 (1.4)
Relapse after HSCT	4 (5.8)
Total	21 (30.3)

Long-term survivors	48 (69.0)

**Table 2:** Outcomes of pediatric acute myeloblastic leukemia of Turkish centers (derived from national thesis database).

Center/publication year/reference	Patients n	Induction death / infection death n(%)	Relapse Rate n(%)	HSCT n(%)	EFS OS
Selçuk University/2009/7	36	4(11%) No data	11 (30.5%)	No data	No data/8y, 35.2%
Uludağ University/2011/8	50	5(10%) 7 (14%)	14(28%)	2(14.2%)	5 y, 52% 60%
Atatürk University/2018/9	74	No data 7 (9.4%)	19 (25.7%)	No data	10 y, 45% 53%
Cerrahpaşa Medical School/2019/10	48	2(4.1%) 11(22.9%)	14(29.2%)	14(29%)	5y, 58.2% 58.2%
Ege University/2019/11	57	2(3.5%) 2 (3.5%)	23(40.4%)	20(35%)	5 y, 50.9% 54%
Present Study/2023	69	4(5.8%) 5 (7.2%)	21(34.4%)	21(34.4%)	15 y, 49.3% 69.6%
